# Identifying and Mapping Ticks on Wild Boars from Romania

**DOI:** 10.3390/ani15081092

**Published:** 2025-04-09

**Authors:** Ioan Cristian Dreghiciu, Mirela Imre, Diana Hoffman, Ion Oprescu, Vlad Iorgoni, Simona Giubega, Sorin Morariu, Marius Stelian Ilie

**Affiliations:** 1 Department of Parasitology and Parasitic Disease, Faculty of Veterinary Medicine, University of Life Sciences “King Mihai I” from Timisoara, 119 Calea Aradului, 300645 Timisoara, Romania; cristian.dreghiciu@usvt.ro (I.C.D.); mirela.imre@usvt.ro (M.I.); diana.hoffman@usvt.ro (D.H.); ioan.oprescu@fmvt.ro (I.O.); simonagiubega@gmail.com (S.G.); sorin.morariu@fmvt.ro (S.M.); 2 Department of Infectious Diseases and Preventive Medicine, Faculty of Veterinary Medicine, University of Life Sciences “King Mihai I” from Timisoara, 119 Calea Aradului, 300645 Timisoara, Romania; vlad.iorgoni@usvt.ro

**Keywords:** wild boar, ticks, SEM, stereomicroscopy, morphology, *Ixodidae*, Romania

## Abstract

Globally, due to climate change, urbanization, and the intensification of interactions between humans and animals, tick populations have increased, and areas where these arthropod vectors can develop and transmit diseases have expanded. *Ixodidae* ticks infect a wide variety of species and serve as major vectors for zoonotic pathogens of veterinary importance. This study aimed to identify and map ticks collected from boar tails in six Romanian counties. A total of 141 ticks were identified and differentiated on the basis of their morphological characteristics via stereomicroscopy and electron microscopy. Among the 141 ticks examined, five species, *Ixodes ricinus*, *Haemaphysalis concinna*, *Haemaphysalis erinacei*, *Dermacentor reticulatus*, and *Dermacentor marginatus*, were identified. The identification and mapping of ticks present on wild boars is beneficial for both veterinary and human medicine due to the pathogens they can transmit. The results of our study indicate that parasitism with different tick species in wildlife—in this case, wild boar—is present in the Romanian counties under study. This is one of the most recent tick identification and mapping studies. Tick parasitism represents a threat to the health of wild/domestic animals, and frequent monitoring is necessary.

## Simple Summary

Ticks are small parasites that can carry and spread diseases to both animals and humans. Because of climate change, growing cities, and closer contact between people and wildlife, tick populations have increased and spread to new areas. In this study, we focused on ticks found on wild boars in six different regions of Romania. We collected and closely examined 141 ticks using special microscopes to identify what types they were. We found five different species of ticks. Knowing which tick species are present on wild animals like boars is important because these ticks can carry harmful diseases. Our research shows that wild boars in the studied regions of Romania are commonly infested with ticks. This information helps veterinarians and public health experts better understand the risks and take steps to protect both animals and people. Regular monitoring of tick populations in wildlife is essential to help prevent the spread of diseases.

## 1. Introduction

The number of tick-borne pathogens (TBPs) and the prevalence of tick-borne illnesses are increasing internationally as a result of multifaceted global changes. Ticks are the primary vectors of disease transmission to people, pets, and livestock [1,2,3]. The environmental elements that influence tick ecology and TBP epidemiology include the quantity and composition of hosts [4,5,6]. Ticks have a greater chance of locating a suitable host, completing their life cycle, and proliferating when the host density is greater [5,7]. Therefore, animals can have a large impact on the epidemiology of TBPs by expanding the range and abundance of ticks and serving as reservoirs for human infections [3,8]. Additionally, when the number of human-wildlife contacts increases in densely populated places, new epidemiological situations arise in which the transmission of zoonotic infections might occur [9,10]. The expansion of suitable habitats has led to increases in tick populations, which may subsequently promote the spread of tick-borne diseases [11]. Ticks may carry many agents at once and are capable of spreading bacterial, parasitic, and viral diseases [12].

The family *Ixodidae*, sometimes referred to as hard ticks, parasitizes a variety of vertebrates, including wild boar. They are distinguished by the presence of a scutum or hard shield. *Ixodidae* ticks can be categorized as endophilic (passive host-finding in burrows) or exophilic (active host-seeking in the environment) species on the basis of how they hunt for hosts. *Ixodes ricinus* is an exophilic species, and all life stages actively quest in the environment; most other *Ixodes* species are endophilic, whereas the genera *Rhipicephalus*, *Dermacentor*, and *Hyalomma* are classified as exophilic. Ticks belonging to *Hyalomma marginatum*, *Rhipicephalus bursa*, and *Dermacentor marginatus* are most frequently observed on wild boars from Mediterranean regions [13].

Although ticks are primarily responsible for the transmission of Lyme borreliosis and tick-borne encephalitis (TBE), ticks have also been identified as vectors of several cases of rickettsiosis, Q fever, babesiosis, anaplasmosis, spotted fever group (SFG) rickettsioses, and tularemia (*Francisella tularensis*) [14].

This study aimed to identify and map ticks on wild boar tails from six counties in Romania.

## 2. Materials and Methods

In the present study, which was carried out between October 2021 and May 2024, ticks were collected from the tails of wild boars found dead or hunted on hunting grounds in the area that was proposed to be surveyed for the presence of parasitic ticks in this category of vertebrate, omnivorous host.

**Figure 1:**
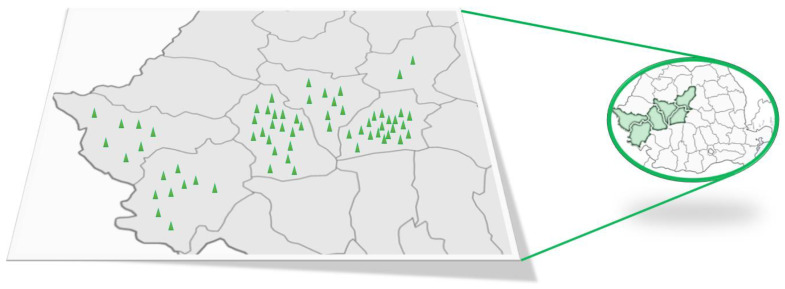
Map of hunting grounds from each county included in the present study (modified from [15]).

The origin areas of the wild boar tails infested with ticks were western and central Romanian counties: Timiș, Caraș-Severin, Hunedoara, Alba, Mureș, and Sibiu (Figure 1). The counties in question boast a rich fauna and a diversified topography, which begins in the west of Romania (Timiș County) with a plain area (the Panonica Plain) and continues to the interior of the Carpathian arc (Hunedoara, Alba, Sibiu, and Mureș Counties), where the topography varies from plains to hills and mountainous areas. The climate is temperate continental transitional, typical of central Europe, with four distinct seasons. Local climatic differences are primarily influenced by altitude and latitude (ANM).

A total of 141 ticks were collected from the tail area of 63 of the 270 boars that were hunted and examined. Tail samples were taken from male and female wild boars aged between 7 and 68 months, and identified and differentiated on the basis of their morphological characteristics via stereomicroscopy (Motic SMZ series, Wetzlar, Germany) and electron microscopy (SEM Hitachi TM3000, Tokyo, Japan) at the Parasitology and Parasitic Diseases Clinic, Faculty of Veterinary Medicine, in Timisoara, Romania [16,17]. The genus *Ixodes* is characterized by a long rostrum and the hypostome is slightly different from species to species, with 2–4 rows of denticles. Pedipalps are elongated; in females, they have different shapes and lengths. The base of the capitulum is species- and sex-specific, with three to six angles and two pairs of posthypostomal hairs in all stages. There are seven differently shaped scutum on the ventral surface in males. The anal siloe unite in the anterior part of the anus or assume a parallel position. Peritremes are round in males and oval in females; the outline is simple without extensions. The coxae are polymorphous with spines and the trochanters have dorsal and sometimes ventral spines. The ventral spine is missing from the extremity of the tarsi. The suckers of the first pair of legs are particularly large in some species. The genus *Dermacentor* is characteristically the easiest to identify. The rostrum is short, with a rectangular base; the palpi are, with some exceptions, short and broad; and the second segment has a conical protuberance entirely covering the first. Pedipalp segments are short and thick. The dorsal shield, capitulum, palp, and extremities have whitish (enamel-like) ornamentation. Males lack adanal spines. The stigmas are round in females and elongated oval in males. The eyes are small and circular or with slightly raised undulations. The coxa of the first pair of legs is bifid in both sexes, and that of the fourth pair is very large in males. The tarsi of all pairs are hooked. The genus *Haemaphysalis* is characterized by a short rostrum; an outwardly prominent second article of the palp; a rectangular capitulum; a retrograde spine in the trochanter of the first pair of legs; 9–10 festoons; no ventral spines in males; oval peritremes; a brownish dorsal shield, uniformly colored with the legs; a relatively small body size; and a lack of eyes and ocelli [16,17].

Every boar was lawfully hunted by licensed hunters. Before being processed, every tick discovered on a single tail of a wild boar was physically removed, put into a separate, 5-milliliter plastic tube, and kept in 70% alcohol. The tubes were labeled by referencing the host—a wild boar. Subsequent analysis identified the species of the ticks collected, their sex (male or female), and their life cycle stage (adult, nymph, or larva). The wild boars’ sex and age class were documented to determine wild boar parasitization (Table 1).

## 3. Results

**Figure 2:**
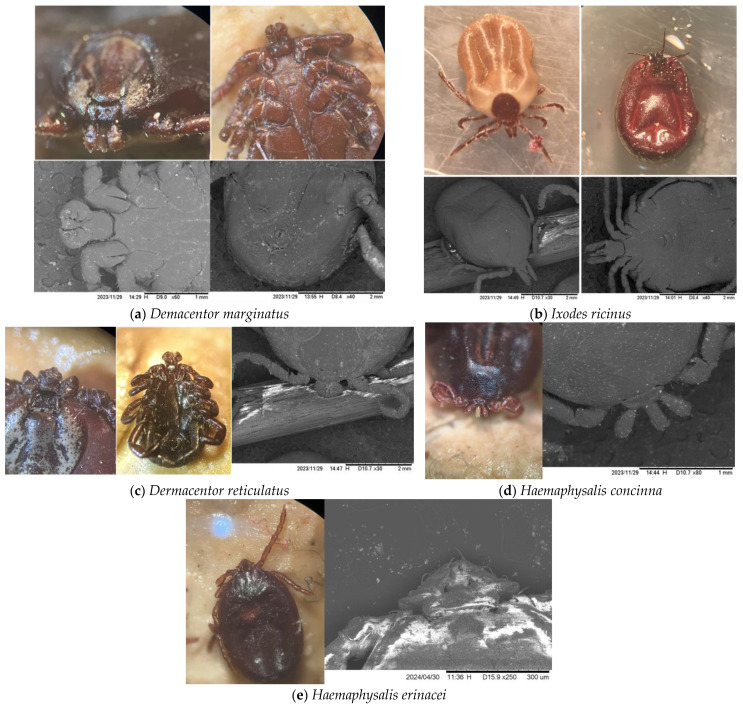
Tick species identified in this research: (**a**) *Dermacentor marginatus*; (**b**) *Ixodes ricinus*; (**c**) *Dermacentor reticulatus*; (**d**) *Haemaphysalis concinna*; and (**e**) *Haemaphysalis erinacei*.

Among the 141 ticks examined, five species present on wild boar tails collected in the six counties were identified on the basis of their morphological characteristics:

- *Dermacentor marginatus* (Figure 2a);
- *Ixodes ricinus* (Figure 2b);
- *Dermacentor reticulatus* (Figure 2c);
- *Haemaphysalis concinna* (Figure 2d);
- *Haemaphysalis erinacei* (Figure 2e).

The gender specificity of *Dermacentor* involves a short rostrum (brevirostrum), ornate scutum, and 11 festoons. The differences between *D. marginatus* and *D. reticulatus* lie in the porous areas (for *D. marginatus*, they are semilunar (oval), and for *D. reticulatus*, they are round) and the spurs of the first coxa (for *D. marginatus*, they are not equal, and for *D. reticulatus,* they are almost equal).

*Ixodes:* In the dorsal view, scapular grooves are present. The palps’ alignment slopes inward. Setae are present on the scutum, and the scutum posterior margin is slightly sinuous. In the ventral view, coxae 1 internal spurs are long, and the genital aperture position is between coxae 4.

*Haemaphysalis*: Palp second articles are broad (in some species they are triangular—ex. *H. erinacei*); festoons are present, but it is unclear when females have fed. Ornamentation is absent from the scutum. Basis capitula has straight lateral margins. The eyes are always absent.

Three variable conditions were analyzed in this study, including the species of ticks, sex, and stage of development (Table 2).

**Figure 3:**
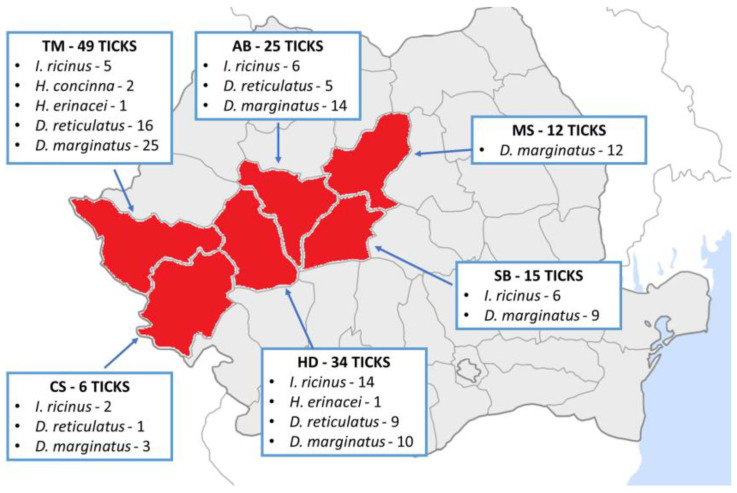
Mapping identified species of ticks in each county (modified after [15]).

As shown on the map (Figure 3), the distribution and mapping of ticks were the variables analyzed for each county under study. In Timiș County, we collected 49 ticks from 91 tails sampled from seven different hunting grounds. In Alba County, 25 ticks from 32 tails from eight hunting grounds were included. In Mureș County, 12 ticks from 17 tails from two hunting grounds were included. In Sibiu County, 15 ticks from 56 tails from 19 different hunting grounds were included. In Hunedoara County, we collected 34 ticks from 58 tails, and in Caraș-Severin, we collected 6 ticks from 16 tails.

In total, 270 tails were examined, where we found the following species mapped by each county studied: in Timiș, there were three genera (*Ixodes*, *Dermacentor*, and *Haemaphysalis*) with five species (*I. ricinus*, *H. concinna*, *H. erinacei*, *D. reticulatus,* and *D. marginatus*), Alba had two genera (*Ixodes* and *Dermacentor*) with three species (*I. ricinus*, *D. reticulatus,* and *D. marginatus*), Mureș had a single species (*D. marginatus*), Sibiu had two genera (*Ixodes* and *Dermacentor*) and two species (*I. ricinus* and *D. marginatus*), Hunedoara had three genera (*Ixodes*, *Dermacentor,* and *Haemaphysalis*) and four species (*I. ricinus*, *H. erinacei*, *D. reticulatus,* and *D. marginatus*), and Caraș-Severin had two genera (*Ixodes* and *Dermacentor*) and three species (*I. ricinus*, *D. reticulatus,* and *D. marginatus*) (Figure 3).

**Figure 4:**
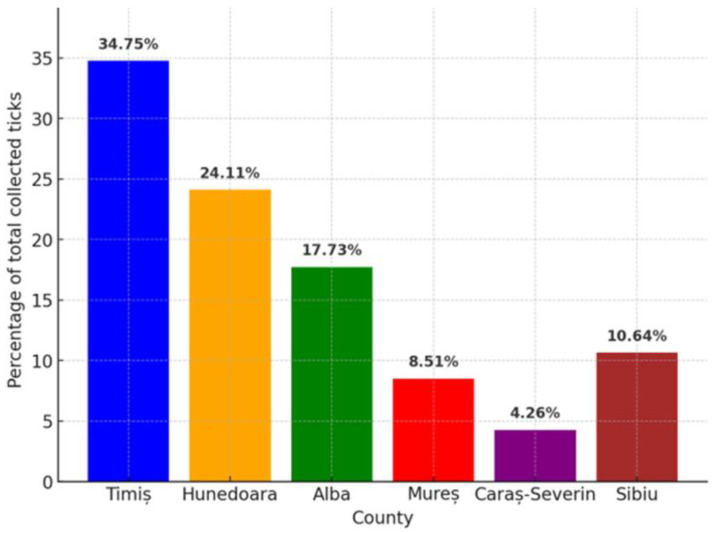
Percentage distribution of collected ticks by county.

The percentage of ticks present on wild boar tails ranged from 12.50% to 34.37%, and the highest prevalence was found in the hunting grounds of Alba County (Figure 4). The results indicate that parasitism by different species of ticks in the wildlife of Romania is present in the studied counties. This study is among the most recent on tick identification and mapping.

## 4. Discussion

The ticks identified on the tails of wild boars in the present study were categorized in three genera and five species, namely, *D. marginatus*, *D. reticulatus*, *H. concinna*, *H. erinacei,* and *I. ricinus*. These genera of ixodid ticks are the most described in terms of distribution in this part of the world and beyond. *Amblyomma*, *Rhipicephalus*, *Dermacentor*, *Haemaphysalis*, *Ixodes,* and *Hyalomma* are the most common genera of ticks that harm both domestic and wild animals [18]. Of course, differences have been found in the distribution and spread of tick species in the different hosts studied in different parts of the world, but it should also be reiterated that ixodid ticks have a very wide host range, which is of particular importance in the epidemiology of tick-borne diseases.

The finding that *D. marginatus* is the most abundant species in the wild boars we studied is not consistent with the majority of studies investigating tick occurrence in Europe, where *I. ricinus* is the most commonly identified species in general, in various host species [19,20,21,22,23]. However, *D. marginatus* in our study, as in other studies on wild boars, was also found to be predominant, supporting this tick–host association [19,24,25,26].

The presence or absence of a tick genus or species in a particular region can be influenced by a number of factors, biotic or abiotic, related to the host species, environment, etc. Biotic and abiotic variables such as host presence, temperature, humidity, and rainfall affect the number of ticks and tick-borne diseases. Furthermore, new infections have emerged worldwide in new environments as a result of the expanding geographic range and incidence of tick vectors and animal reservoir hosts [1].

*Dermacentor reticulatus* has been identified as the third most abundant species in wild boars under study, and it is, in fact, the second most commonly reported tick species in Central Europe, after *Ixodes ricinus* [27].

Among the members of the genus *Haemaphysalis*, *H. concina* and *H. erinacei* were detected in wild boars in our study. *Haemaphysalis* parasitize a wide range of hosts in their immature stage and only mammals in their adult stage. Our results are somewhat different from those obtained in other studies in which *H. adleri*, *H. erinacei*, *H. parva*, *H. punctata*, and *H. sulcata* have been identified in wild animals in Cyprus, Greece, Israel, Italy, Slovenia, Spain, Turkey, and France [19,28].

The role of wild boars as reservoir hosts for multiple tick-borne pathogens is well documented, yet under specific ecological conditions, they may also act as dilution hosts. The impact of wild boars varies by region, pathogen, and tick species, making them a significant subject in the study of tick-borne disease ecology. Wild boars serve as a significant host for ticks and tick-borne pathogens (*Anaplasma phagocytophilum* [29], *Babesia* cf. *crassa* [30], *Rickettsia monacensis* [31], *Babesia divergens* [29], and *Rickettsia* spp. [32]) due to their large size, habitat, and longevity. They are among the most widespread mammals globally, particularly as suids. While some populations have been affected by African swine fever, they remain overabundant in many regions, including Europe. Their long life, migratory behavior, varied feeding habits, and extensive distribution make them effective sentinel species for monitoring the spread and distribution of pathogens and ixodid ticks [31]. The ongoing discovery of new tick-borne infections has led to a dynamic epidemiology and diversity of recognized vector-borne illnesses and pathogens [33]. Studies on tick dispersion are essential because they provide information on the dissemination of pathogens carried by ticks and the environmental factors that affect them [30].

In the Balkan region, eight animal hosts were identified as infected, with the primary five wild animals being red deer, rodents, wild boars, roe deer, and birds. A total of 16 tick species from six genera were reported from these hosts. The predominant genera were *Hyalomma* spp., followed by *Rhipicephalus* spp., *Haemaphysalis* spp., and *Ixodes* spp. [28].

In research conducted in Hungary, the majority of the samples were adult ticks from six species and nymphs: *I. ricinus* (43.2%), *I. canisuga* (5.6%), *D. reticulatus* (48.9%), *H. concinna* (2%), *D. marginatus*, and *I. hexagonus*. The species that were recognized the most frequently were *D. reticulatus* and *I. ricinus* [34].

In Poland, *D. reticulatus* was in noticeably greater abundance in the studied areas [35,36].

Ticks collected in Italy were identified as *D. marginatus*, *Rh. bursa*, and *H. sulcata* [34,37]. Additionally, animals were infected with multiple tick species, including *H. erinacei*, *I. hexagonus*, *R. turanicus*, *I. canisuga*, *I. ricinus*, *R. bursa*, and *D. marginatus*. Among all the tick species found, *R. turanicus* had the widest host range [38].

A total of 495 adult ticks—60% female and 40% male—were collected in France. At various collection locations, three species of ticks were found: *D. marginatus* (*n* = 377; 76%), *D. reticulatus* (*n* = 74; 15%), and *I. ricinus* (*n* = 45; 9%) [39].

In a review by Defaye et al. (2022) [28] focusing on the Mediterranean Rim in Western Europe, it was noted that in the literature on pathogens in wild animals, ticks were primarily found in France, Italy, and Spain. Of the 17 reported animal hosts, 16 were identified in Western Europe, with red deer (32%), wild boars (27%), rodents (26%), birds (25%), and roe deer (17%) being the most frequently included. The review identified 31 tick species associated with these hosts across the region, categorized into eight genera: *Ixodes*, *Hyalomma*, and *Dermacentor*, which primarily affect ungulates, and *Argas* spp., *Amblyomma* spp., *Haemaphysalis* spp., *Ornithodoros* spp., and *Rhipicephalus* spp. [28].

The geographical distribution of *D. marginatus* and *D. reticulatus* in different countries in Europe, such as Austria (4/6) [40,41,42,43], Belarus (0/32) [44], Belgium (0/25) [45], Bulgaria (5/0) [46,47,48,49,50], Germany (83/115) [51,52,53,54], Great Britain (0/2) [55], Greece (26/0) [56], Hungary (18/54) [57,58,59], Italy (35/0) [60,61,62], Latvia (0/12) [63], Lithuania (0/66) [64], the Netherlands (0/51) [65,66], Poland (0/114) [36,67,68,69,70,71,72,73], Portugal (6/7) [74,75], Romania (433/68) [76,77], Serbia (4/8) [78], Slovakia (14/71) [79,80,81], Switzerland (33/13) [82,83,84], Turkey (16/0) [85,86,87,88], and Ukraine (15/61) [89,90], was presented by Rubel et al. [91].

In Belarus, 553 ticks of the species *Ixodes ricinus* (59.1%; 327/553) or *D. reticulatus* (40.9%; 226/553) were taken from vegetation (81.9%; 453/553), cattle (17.9%; 99/553), or dogs (0.2%; 1–553). While *D. reticulatus* was taken from cattle in the majority of cases (61.6%; 61/99), *I. ricinus* made up the majority of ticks retrieved from vegetation (63.8%; 289/453) [44].

In a study conducted in all six districts of Cyprus, 3057 adult ticks from 11 species and four genera were extracted from 441 (24.6%) infected animals. The most common species was *R. sanguineus* (1176 ticks, 38.5%), followed by *R. turanicus* (651 ticks, 21.3%) and *R. bursa* (544 ticks, 17.8%). The frequency of *Hyalomma excavatum* and *Haemaphysalis sulcata* varied, with 276 ticks (9.0%) each. The remaining 4.4% was made up of *I. gibbosus*, *H. marginatum*, *H. rufipes*, *I. ventalloi*, *H. punctata*, and *R. pusillus*. A total of 1296 ticks from ten different species, including *R. bursa* (210 ticks), *H. sulcata* (276 ticks), and *R. turanicus* (175 ticks), were found infesting wild animals [92].

In the context described above, the geographical distribution and host diversity of tick species play a very important role in surveillance and risk assessment for both humans and animals affected by tick-borne diseases, such as tick-borne encephalitis, Lyme borreliosis, or rickettsiosis. This study contributes to the knowledge in this field, providing information on the role of wild boars in the spread of vectors and pathogens [93].

## 5. Conclusions

In conclusion, the identification and mapping of ticks present on wild boars are beneficial for both veterinary and human medicine due to the pathogens they can transmit. In this study of tick prevalence (23.33%) in wild boars raises concern about the diseases that may exist in them. Tick parasitism represents a threat to the health of wild/domestic animals, and frequent monitoring is necessary.

## Figures and Tables

**Table 1:** Distribution of tails and ticks by county.

County	Hunting Grounds Total/Positive/%	Tails Sampled			% Ticks Found on Tails
		Total	With Ticks		
			No. (%)	Average No. Ticks/Tail	
Timiș	18 (7) 38.89%	92	25 (27.174)	1.96	34.75% (49/141)
Hunedoara	20 (9) 45%	57	13 (22.807)	2.62	24.11% (34/141)
Alba	9 (6) 66.67%	32	11 (34.375)	2.27	17.73% (25/141)
Mureș	2 (2) 100%	16	4 (25)	3	8.51% (12/141)
Caraș-Severin	10 (3) 30%	16	3 (18.750)	2	4.26% (6/141)
Sibiu	21 (6) 28.57%	56	7 (12.5)	2.14	10.64% (15/141)
Total	80 (33) 41.25%	270	63 (23.333)	2.24	141

**Table 2:** Variable conditions of analyzed ticks.

Epidemiological Factors	No. of Ticks	(%)
Sex		
Male	37	26%
Female	104	74%
Stage of development		
Adult	141/141	100%
Species		
*Ixodes ricinus*	33	23%
*Dermacentor reticulatus*	31	21%
*Dermacentor marginatus*	73	52%
*Haemaphysalis concinna*	2	2%
*Haemaphysalis erinacei*	2	2%

## Data Availability

Data are contained within the article.
